# Trio-Based Whole-Exome Sequencing Identifies Convergent Epithelial Junction-Related Pathways in Syndromic Hidradenitis Suppurativa

**DOI:** 10.1159/drm/ackag016

**Published:** 2026-09-08

**Authors:** Chiara Moltrasio, Paola M. Tricarico, Elena M. Nardacchione, Ronald Moura, Sara Roncarà, Francesca Satolli, Flavia Manzo Margiotta, Valentina Dini, Angelo V. Marzano, Sergio Crovella

**Affiliations:** a Dermatology Unit, Fondazione IRCCS Ca’ Granda Ospedale Maggiore Policlinico, Milan, Italy; b Pediatric Department, Institute for Maternal and Child Health - IRCCS - “Burlo Garofolo”, Trieste, Italy; c Department of Medical Surgical and Health Sciences, University of Trieste, Trieste, Italy; d Department of Advanced Diagnostics, Institute for Maternal and Child Health - IRCCS - “Burlo Garofolo”, Trieste, Italy; e Dermatology Unit, Department of Medicine and Surgery, University of Parma, Parma, Italy; f Department of Dermatology, University of Pisa, Pisa, Italy; g Health Science Interdisciplinary Center, Sant’Anna School of Advanced Studies, Pisa, Italy; h Department of Pathophysiology and Transplantation, Università degli Studi di Milano, Milan, Italy; i Department of Environmental and Prevention Science, University of Ferrara, Ferrara, Italy

**Keywords:** Syndromic hidradenitis suppurativa, Whole-exome sequencing, Pathway analysis, Cell junction-related defects, Epidermal barrier impairment

## Abstract

**Introduction:**

Hidradenitis suppurativa (HS)-related autoinflammatory syndromes, simply termed syndromic HS (sHS), represent a group of rare immune-mediated inflammatory disorders in which HS coexists with systemic or cutaneous autoinflammatory features like PASH (pyoderma gangrenosum [PG], acne, and HS), PAPASH (PASH, pyogenic arthritis), PASS (PG, acne, HS, and ankylosing spondylitis), and SAPHO syndrome (synovitis, acne, pustulosis, hyperostosis, and osteitis). In recent years, genetic studies identified several novel pathogenic variants underlying sHS; however, most investigations rely exclusively on affected individuals’ sequencing, and the absence of parental genomic information limits the possibility of determining inheritance patterns.

**Methods:**

This study aimed to address these gaps; we performed trio-based whole-exome sequencing on five individuals diagnosed with sHS and their unaffected parents.

**Results:**

The pathway related to epidermal adhesion and desmosome organization was the most represented across our cohort, encompassing seven genes: *DSC3, DSG1, FAT1, LAMA3, MICALL2, PLEC*, and *TJP2*. Integrin-extracellular matrix (ECM) adhesion signaling pathway, represented by ten genes (*CSPG4, FERMT3, ITGA3, LAMA3, LAMA5, LIMS2, LTBP3, PLEC, TGM2,* and *TNC*), was also retrieved. Also, variants affecting innate immune pathways, including cytokine signaling and antigen presentation, have been observed.

**Conclusion:**

Our exploratory findings suggest that genetically heterogeneous variants in syndromic HS converge on biological processes involving epithelial junction organization, ECM interactions and innate immune regulation. Although not establishing a unique pathogenic mechanism, these observations identify epithelial barrier biology as a candidate pathway warranting validation in larger cohorts and functional studies.

## Introduction

Hidradenitis suppurativa (HS) is a complex autoinflammatory skin disorder of the terminal hair follicle, manifesting with painful, deep-seated nodules, abscesses, draining tunnels and hypertrophic scarring in apocrine gland-bearing skin [1]. Besides common comorbidities, in a rare subset of patients, HS may coexist with other immune-mediated inflammatory diseases or inherited conditions, defining the clinical setting of HS-related autoinflammatory syndromes or syndromic HS (sHS) [2–4]. The clinical triad of pyoderma gangrenosum (PG), acne, and suppurative hidradenitis (PASH) represents the prototype of sHS [5], together with PAPASH (pyogenic arthritis, acne, PG and HS) and PASS (PG, acne, HS and spondyloarthritis) [2]. Synovitis, acne, pustulosis, hyperostosis, and osteitis (SAPHO) is another autoinflammatory condition in which HS represents a common dermatological manifestation [6, 7].

Although HS is traditionally considered a postpubertal disease with a low prevalence in the pediatric population – estimated at approximately 3.2% in patients under 10 years of age – the pediatric perspective is increasingly recognized as a crucial clinical frontier [8, 9]. In sHS, pediatric onset is not a rare manifestation but frequently represents a defining feature of a distinct autoinflammatory phenotype. This early presentation is often characterized by heightened inflammatory activity, atypical clinical trajectories, and increased disease severity, which together necessitate heightened diagnostic vigilance in childhood. Recognizing this phenotype at an early stage is critical, as delayed diagnosis may result in prolonged systemic inflammation and cumulative morbidity. Accordingly, timely identification should prompt age-appropriate, phenotype-specific management strategies that account for developmental considerations, long-term disease course, and the unique therapeutic needs of pediatric patients. sHS patients, both children and adults, suffer a profound negative impact on their quality of life due to extensive skin involvement, frequent association with systemic comorbidities and, in most cases, refractoriness to treatments, causing additional diagnostic and therapeutic challenges [2, 3].

A range of genetic defects have been involved in sHS pathogenesis, including pathogenic variants in genes related to the gamma-secretase complex such as Nicastrin (*NCSTN*) as well as in auto-inflammatory-related genes [10, 11]. Additionally, genetic changes closely related to vitamin D metabolism and keratinization pathways have been detected [12].

Despite recent advances in defining the genetic landscape of sHS, most studies have relied on sequencing affected individuals only, limiting the ability to determine variant origin. Trio-based sequencing overcomes this limitation by enabling the identification of de novo and inherited variants, an important advantage in genetically heterogeneous disorders dominated by private mutations. Given the rarity and marked heterogeneity of sHS, our objective was not to identify recurrent disease-causing genes, but to determine whether genetically distinct cases converge on common biological processes. Accordingly, we designed this study as an exploratory, hypothesis-generating investigation integrating inheritance-aware whole-exome sequencing (WES) with pathway-oriented biological interpretation to identify candidate mechanisms for future validation in larger cohorts.

## Methods

### Patients

Three Italian dermatology units contributed to the collection of clinical data and saliva samples from sHS patients and their parents. Genetic counseling was also performed.

### DNA Extraction, WES, and Data Analysis

Saliva samples were collected using ORAgene DNA kits (OG-500), and DNA was extracted following the manufacturer’s instructions. WES was outsourced and performed by Macrogen Europe (Amsterdam, The Netherlands). The Exome Sequencing Analysis, with a declared 150× coverage, used the Illumina SureSelect Human V7 Kit Library preparation and sequencing reaction, on an Illumina NovaSeq X Sequencing, generating pair-end reads of 150 base pairs. Quality control of the fastq.gz input was performed before the procedures using multiqc v.1.31 [13]. Library adapters and reads with low-quality scores (<20) have been removed using the fastp v.1.0.1 application [14]. The aligning was performed using the Burrows-Wheeler v.0.7.19 Aligner software package [15] using the most recent Reference Human Genome version (GRCh.38p14). Next, the Picard toolkit v.3.4.0 [16] was used to remove and mark duplicated reads, and GATK v. 4.6.0 [17], for base quality score recalibration in accordance with established best-practice guidelines. Variant calling was carried out using Strelka2 v.2.9.10 [18]. Variants were prioritized according to quality, variant type, rarity, inheritance, and biological plausibility. Given the rarity and genetic heterogeneity of sHS, the study aimed to identify biologically plausible candidate variants for inheritance-aware interpretation rather than recurrent pathogenic genes. To ensure the robustness and reliability of variant calls, we applied genotype-level quality filters. Specifically, we retained only variants supported by an allele depth greater than 10 reads, thereby minimizing the impact of low-coverage artifacts. Genotype quality was required to exceed 20, reflecting a high confidence in the assigned genotype. Variant annotation was performed with ANNOVAR software [19]. Variants were retained only if they were splicing or exonic non-synonymous variants and had an allele frequency of <0.01 or were absent from the Genome Aggregation Database [20].

Variants with a Combined Annotation Dependent Depletion (CADD) score of >15 were considered supportive of potential deleteriousness [21]. Variants lacking CADD annotation were evaluated according to predicted molecular consequence, rarity, inheritance pattern and biological plausibility rather than retained because of the absence of a CADD score. Additionally, to ensure biological relevance to the disease context, gene expression data from the Genotype-Tissue Expression (GTEx) project [22] were evaluated. GTEx skin expression was incorporated as a biological prioritization criterion after quality filtering and inheritance-based variant classification to facilitate interpretation within the disease-relevant tissue. Although not intended to infer pathogenicity, this approach may underrepresent genes acting through systemic immune or developmental pathways, a limitation considered when interpreting the results.

Each variant was evaluated under three inheritance hypotheses: (i) heterozygous de novo mutations present exclusively in the proband; (ii) homozygous from two heterozygous carrier parents; and (iii) compound heterozygous configurations where two distinct pathogenic alleles were inherited, one from each parent. Prioritized genes were functionally annotated using KEGG and Reactome pathway databases. Given the exploratory design and limited cohort size, pathway-level findings were interpreted as descriptive evidence of biological convergence rather than formal statistical enrichment. Since all the syndromic conditions examined share HS as the common dermatological phenotype, we directed attention to pathways previously implicated in HS pathophysiology [23].

## Results

### Clinical Features

The main clinical features of five probands diagnosed with sHS are shown in Table 1. Four patients, 1 of whom had a form overlapping with SAPHO, were affected by PASH and 1 by PAPASH. One PASH patient suffered from concomitant inflammatory bowel disease and osteoporosis, while another developed paradoxical psoriasis. HS onset occurred at a mean age of 20.2 years. Notably, 60% of the cohort exhibited a pediatric onset, as documented during anamnesis at the time of diagnosis (HSM404: onset at 13 years; HSM25: onset at 14 years; HSM22: onset at 15 years; Table 1). Although disease manifestation frequently preceded or coincided with puberty, the mean age at diagnosis was 24.6 years, indicating a substantial diagnostic delay. This delay was markedly longer in patients with pediatric onset: 13 years in the individual with disease onset at 14 years (diagnosed at 27 years) and 7 years in the patient with onset at 13 years (diagnosed at 20 years). In contrast, the patient with adult-onset disease experienced minimal diagnostic latency, with diagnosis occurring 1 year after symptom onset (onset at 41 years, diagnosis at 42 years) (Table 1).

**Table 1.** Clinical characteristics of patients diagnosed with HS-related autoinflammatory syndromes

Patient ID/Sex/Diagnosis	Comorbidities	HS			PG			Acne			Main systemic treatments/surgery	Clinical course at last follow-up
		age at onset	age at diagnosis	IHS4 at diagnosis	age at onset	age at diagnosis	PG score at diagnosis	age at onset	age at diagnosis	GAG score at diagnosis		
HSM5/M/PASH	IBD; osteoporosis	18	18	23	19	19	Multilesional	16	16	31	Azitromicine; trimetoprim-sulfametoxazole; ceftriaxone; ciprofloxacine; hymecycline; rifampicin; doxycyclin; methylprednisolone; dapsone; ciclosporine; adalimumab; anakinra; ustekinumab; bilateral surgery	Partial response
HSM22/M/PASH/SAPHO overlapping	SAPHO	15	16	26	15	16	Multilesional	13	13	28	Rifampicin; doxycycline;azitromycine; hymecycline;clindamycin; intravenous immunoglobulin; methotrexate; isotretinoin; deltacortene	Partial response
											adalimumab; anakinra; ustekinumab; secukinumab	
HSM25/M/PAPASH	Pyogenic Arthritis	14	27	30	18	27	Multilesional	13	14	32	Rifampicin; doxycycline; azitromycine; hymecycline; clindamycin; prednisone; adalimumab; anakinra; acitretin; guselkumab; secukinumab; infliximab; povorcitinib; upadacitinib	Partial response
HSM404/M/PASH	n/a	13	20	30	18	13	Multilesional	16	16	32	Dodycycline; acitretin	Partial response
											ciclosporine	
											adalimumab	
											secukinumab	
HSM426/F/PASH	Paradoxical psoriasis	41	42	29	41	41	Multilesional	14	14	28	Clindamycin; cyclosporine; dapsone; doxycycline; prednisone; adalimumab; ixekizumab; bimekizumab	Partial response

PASH, pyoderma gangrenosum, acne, hidradenitis suppurativa; PAPASH, pyogenic arthritis, pyoderma gangrenosum, acne, hidradenitis suppurativa; SAPHO, synovitis, acne, pustulosis, hyperostosis, and osteitis; PG, pyoderma gangrenosum; IBD, inflammatory bowel disease; IHS4, International Hidradenitis Suppurativa Severity Score System 4; GAG, Global Acne Grading System.

The disease was chronic-relapsing in all cases, with an average IHS4 (International Hidradenitis Suppurativa Severity Score System 4) [24] value at diagnosis of 27.6. PG was ulcerative and multilesional in all cases, presenting at a mean age of 22.2 (parallel to the age at HS onset with a mean difference between them of 2 years). Similarly, mean age at PG diagnosis was 23.2 with a mean difference of 1.4 years compared to the age at HS diagnosis.

Notably, all patients exhibited a pediatric onset of acne (mean age of 14.4), with a comparable mean age at diagnosis of 14.6 years, prior to the manifestation of HS/PG. This early presentation was characterized by a mean Global Acne Grading (GAG) score of 30.2, indicating moderate-to-severe involvement.

All patients were initially treated with conventional systemic approaches, including systemic antibiotics and high-potency corticosteroids for PG lesions. All patients were subsequently treated with biological agents, including tumor necrosis factor antagonists and inhibitors of interleukin (IL)-1, IL-17 and IL-23. In 1 PAPASH patient (HSM25), JAK-STAT inhibitors povorcitinib and upadacitinib, were administered. Bilateral surgery for HS was performed in 1 PASH patient (HSM5).

### Genetic Findings

As shown in Figure 1, functional annotation identified no biological process common to all 5 patients. Three pathways were recurrently altered in four individuals:(i) cell junctions, including desmosomal organization (HSM22, HSM404, HSM426, HSM5); (ii) innate immune activation through cytokine-mediated, Toll-like receptor, NOD-like receptor, and downstream NF-κB signaling (HSM22, HSM25, HSM426, and HSM5), and (iii) antigen processing and presentation via MHC class I-II molecules (HSM22, HSM25, HSM404, and HSM426). The pathway related to epidermal adhesion and desmosome organization was the most strongly represented, encompassing seven genes (Fig. 1). Additional pathways exhibited partial overlap: immunometabolic and energy-regulation processes (HSM22, HSM404, and HSM426), and Notch signaling (HSM25, HSM404, and HSM5), were identified in three individuals, while integrin-extracellular matrix (ECM) adhesion (HSM404 and HSM426) and sphingolipid metabolism perturbations (HSM404 and HSM426) appeared in two. A broader pathway analysis integrating all genes identified across the cohort further supported the centrality of epithelial adhesion mechanisms in sHS. Two biological processes were consistently represented across the prioritized genes: integrin-ECM adhesion signaling – represented by ten genes (*CSPG4, FERMT3, ITGA3, LAMA3, LAMA5, LIMS2, LTBP3, PLEC, TGM2*, and *TNC*) – and epidermal cell-cell junction and desmosomal organization – supported by seven genes (*DSC3, DSG1, FAT1, LAMA3, MICALL2*, *PLEC,* and *TJP2*).

**Fig. 1.**
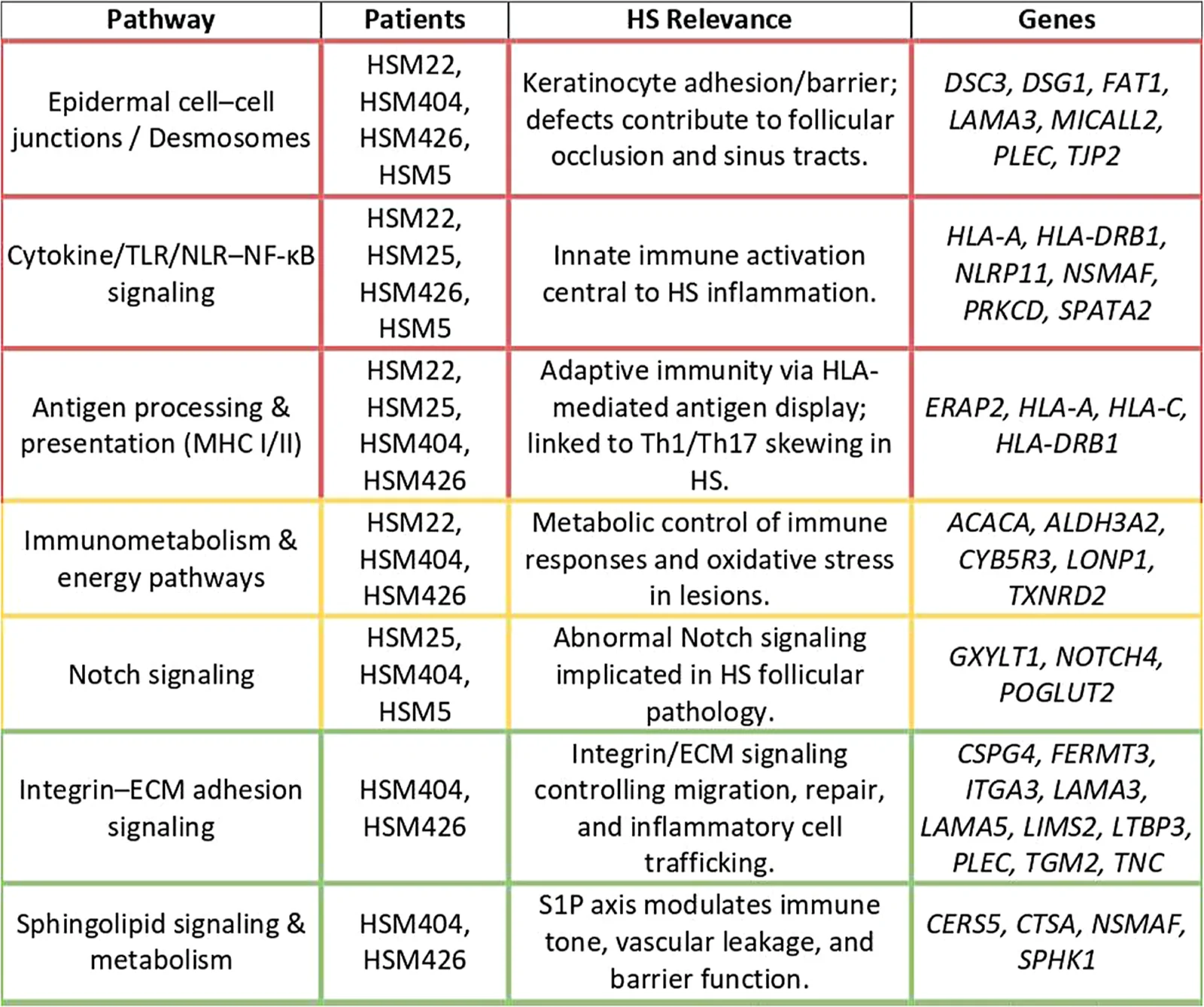
Functional pathway annotation integrating all prioritized genes identified across the cohort. Epidermal adhesion (including desmosome organization), innate immune activation through cytokine-mediated, Toll-like receptor, NOD-like receptor, and downstream NF-κB signaling and antigen processing and presentation via MHC class I–II molecules were the most represented pathways. Additional pathways such as immunometabolic and energy-regulation processes, Notch signaling, integrin-extracellular matrix (ECM) adhesion and sphingolipid metabolism perturbations were also retrieved.

## Discussion

We performed trio-based WES in 5 patients with syndromic HS to explore the biological basis of this genetically heterogeneous disorder. Although no recurrent disease-causing gene was identified, independently prioritized rare variants converged on pathways involving epithelial junction organization, ECM interactions and innate immune regulation, providing candidate mechanisms for future genetic and functional studies. While the small sample size precludes genotype-phenotype correlations, these findings reinforce the importance of improved molecular characterization to support earlier diagnosis and optimized clinical management.

Epidermal junctions mediate both extracellular adhesion between neighboring cells and intracellular anchoring to distinct components of the cytoskeleton [25]. Cell junction proteins are not only exclusively associated with mechanical adhesion, but their main role is to maintain homeostasis by regulating structural tissue integrity. Furthermore, junctions in the epidermis and hair follicles have been shown to be involved in communication between adjacent cells with diffusion of ions and solutes, in cell proliferation, cell migration and apoptosis [26–28].

A growing body of evidence now suggests that the multifaceted role of epithelial junctions extends far beyond anchorage-dependent biological processes or those related to changes in cell shape. These roles converge on the regulation of innate immune defense and cellular stress resistance. In particular, junctional complexes act as upstream regulatory platforms that integrate environmental and intracellular signals to modulate epithelial junction organization, barrier integrity, and permeability. Through these mechanisms, they influence multiple aspects of epithelial barrier function, including pathogen sensing, inflammatory signaling, and adaptive responses to mechanical and metabolic stress [29].

In recent years, the central role of epithelial barrier dysfunction in the etiopathogenesis of a broad spectrum of inflammatory skin disorders has been reported [30–32]. These findings establish a link between the regulation of epidermal barrier integrity and the onset, progression, and persistence of cutaneous inflammation, thereby highlighting barrier homeostasis as a critical determinant in the therapeutic management of chronic inflammatory skin diseases.

HS is a heterogeneous disorder characterized by autoinflammation, dysregulation of the innate and adaptive immune system and alteration in keratinization processes [1, 10–12]. Recent results from genome-wide association studies have identified significant genetic variants in *SOX9*, *INAVA* and *KLF5* genes associated with HS, providing strong evidence for a genetic contribution to disease susceptibility and implicating these genes in key pathways underlying HS pathogenesis [33]. While SOX9 is an essential transcription factor for hair follicle formation and epithelial stem cell fate, *INAVA* and *KLF5* are associated with epithelial barrier integrity [34]. It has also been recently hypothesized that *KLF4,* a zinc finger-containing transcription factor that regulates several cellular processes in barrier formation, may influence HS pathogenesis [35].

Our genetic analyses have revealed that the majority of disease-associated variants identified in *PSTPIP1*, *MEFV*, and *NOD2* are private mutations, underscoring marked interindividual genetic heterogeneity. Notably, these loci exhibit significant overlap with genes implicated in other autoinflammatory disorders [10, 33].

Also, our results fit within the current structural model of HS pathogenesis. Danby et al. [36] proposed that defective mechanical support of the follicular unit may represent an initiating event leading to follicular rupture and secondary inflammation rather than inflammation being the primary event. Consistent with this model, Nelson et al. [37] demonstrated loss of E-cadherin and p120-catenin expression in HS lesions, providing experimental evidence that disruption of epithelial junctions accompanies disease development.

To discuss a few representative examples, LAMA3 (laminin subunit alpha 3) is a key component of the epithelial basement membrane and is responsive to multiple epithelial–mesenchymal regulatory cues, including keratinocyte-derived and epidermal growth factors. Through its role in cell-matrix interactions and epithelial integrity, LAMA3 has emerged as an important molecular target in the study of skin homeostasis and inflammatory skin diseases [36]. In parallel, DSC3 (desmocollin-3) and DSG1 (desmoglein 1) are desmosomal cadherins that are essential for stable cell-cell adhesion and epidermal cohesion. Dysregulation of these molecules compromises desmosomal integrity and has been predominantly implicated in autoimmune and inflammatory skin disorders, underscoring their relevance in epidermal barrier dysfunction [38].

This suggests that sHS may share a fundamental pathogenic paradigm with other inherited barrier disorders, such as atopic dermatitis, epidermolysis bullosa and autoimmune blistering diseases, in which genetic disruption of structural components leads to mechanical fragility and chronic inflammation [39]. Interestingly, convergence on genes involved in epithelial adhesion and barrier organization parallels findings in several inherited and inflammatory skin disorders, including atopic dermatitis, epidermolysis bullosa and autoimmune blistering diseases [40]. Furthermore, recent evidence identified previously unreported junctional abnormalities in apocrine glands, where defects weaken the follicle-gland unit and trigger a subsequent foreign body–type immune response [41]. Although not implying a shared pathogenic mechanism, this observation supports epithelial barrier dysfunction as a candidate biological process in syndromic HS.

In conclusion, this exploratory trio-based WES study suggests that genetically heterogeneous variants in syndromic HS converge on common biological processes involving epithelial junction organization, ECM interactions and innate immune regulation. These findings highlight epithelial barrier biology as a candidate pathway for future investigation, warranting validation in larger multicenter cohorts integrating inheritance-aware genomics and functional studies. From a clinical perspective, improved molecular characterization may facilitate earlier diagnosis and more refined patient stratification, particularly in pediatric and early-onset disease.

## Statement of Ethics

This study protocol was reviewed and approved by Comitato Etico Unico Regionale of Friuli Venezia Giulia, Institute for Maternal and Child Health “IRCCS Burlo Garofolo,” Approval No. CEUR-2018-Sper-127-BURLO). All participants signed a written informed consent.

## Conflict of Interest Statement

The authors have no conflicts of interest to declare.

## Funding Sources

This work was supported by the Italian Ministry of Health, through the contribution given to the Institute for Maternal and Child Health IRCCS Burlo Garofolo, Trieste (Italy), and by the “ENIGMA” Project funded by EADV (No. PPRC-2023-0047). This research was also partially supported by the Italian Ministry of Health (Ricerca Corrente) of Fondazione IRCCS Ca’ Granda Ospedale Maggiore Policlinico, Milan (Italy). The funders had no role in the design, data collection, data analysis, and reporting of this study.

## Author Contributions

Conceptualization and data curation: C.M., P.M.T., and S.C.; formal analysis: P.M.T., E.M.N., R.M., S.R., and S.C.; funding acquisition: P.M.T. and A.V.M.; investigation C.M., P.M.T., E.M.N., F.S., F.M.M., V.D., and S.C.; methodology: S.R., F.S., F.M.M., V.D., and R.M.; project administration P.M.T. and S.C.; resources C.M., P.M.T., A.V.M., and S.C.; software R.M. and S.C.; supervision P.M.T., A.V.M., and S.C.; Validation: C.M., P.M.T., and S.C.; visualization P.M.T. and S.C.; writing – original draft preparation: C.M., P.M.T., E.M.N., and S.R.; writing – review and editing: C.M., P.M.T., E.M.N., S.R., and S.C.

## Data Availability

The data that support the findings of this study are not publicly available due to privacy reasons but are available from the corresponding author upon request.
